# Jingangteng Capsule Attenuates Ulcerative Colitis via Maintaining the Homeostasis of Intestinal Microbiota and Metabolites, Inhibiting the PI3K-AKT-mTOR Signaling Pathway

**DOI:** 10.3390/ph19040589

**Published:** 2026-04-07

**Authors:** Jing Li, Yue Xiong, Shiyuan Cheng, Dan Liu, Qiong Wei, Xiaochuan Ye

**Affiliations:** 1 Hubei Key Laboratory of Resources and Chemistry of Chinese Medicine, School of Pharmacy, Hubei University of Chinese Medicine, Wuhan 430065, China; 2 Hubei Shizhen Laboratory, Hubei University of Chinese Medicine, Wuhan 430065, China

**Keywords:** Jingangteng capsule, male BALB/c mice, ulcerative colitis, gut flora, metabolome, PI3K-AKT-mTOR

## Abstract

**Background/Objectives**: Ulcerative colitis (UC) involves inflammatory response, oxidative stress, changes in metabolites, and the gut microbiota. Jingangteng capsule (JGTC) has been utilized clinically for the treatment of inflammatory diseases for many years. However, the efficacy of JGTC in ameliorating UC remains unclear, and the underlying mechanisms have not yet been elucidated. This study aims to investigate the effect and mechanism of JGTC on UC. **Methods**: The chemical compositions of JGTC were examined using ultra-high-performance liquid chromatography with quadrupole time-of-fight mass spectrometry. The anti-UC effect of JGTC was evaluated by assessing the disease activity index (DAI), colon length, intestinal barrier recovery, and inflammatory factors in a dextran sulfate sodium (DSS)-induced colitis model. Mechanisms were investigated through fecal 16S rDNA sequencing, metabolomics analysis, enzyme-linked immunosorbent assay (ELISA), Western blotting, and network pharmacology analysis. **Results**: JGTC significantly reduced the DAI scores in UC mice, increased their body weight and colon length (*p* < 0.001), repairing damaged intestinal tissue. It decreased the levels of inflammatory cytokines TNF-α, IL-6, IL-1β, and LPS (*p* < 0.01, *p* < 0.001), alleviating intestinal inflammation. It also raised the expression of tight junction proteins ZO-1, Claudin-1, and Occludin (*p* < 0.05, *p* < 0.001), thereby enhancing intestinal barrier function. Fecal metabolomic analysis revealed that the favorable alterations in amino acid and lipid metabolites were more pronounced. Heat maps showed strong correlations between pharmacological indicators and gut microbiota, as well as between the main differential metabolites and gut microbial communities. UPLC-QTOF-MS detection yielded 33 components of JGTC, and network pharmacology analysis based on these components predicted pathways of action of JGTC in UC. Functional pathways closely associated with significantly differential metabolites and metabolic pathways were also investigated. The PI3K-AKT-mTOR pathway was one of them, which is consistent with the conclusions drawn from network pharmacology. JGTC significantly modulated key factors in this pathway, inhibiting the expression of PI3K, Akt, PDK1, and mTOR, while augmenting the expression of PTEN (*p* < 0.05, *p* < 0.01, *p* < 0.001). It also mitigated the levels of related oxidative stress factors MDA, MPO, and D-LA, and raised SOD levels (*p* < 0.01, *p* < 0.001). **Conclusions**: JGTC improved the excessive inflammatory response in UC by regulating intestinal flora and metabolic disorders, affecting the PI3K-AKT-mTOR signaling pathway, restoring intestinal tissue damage and intestinal barrier, and inhibiting inflammatory and oxidative stress factors.

## 1. Introduction

Ulcerative colitis (UC) is a chronic inflammatory disease. Patients present with intestinal inflammation and destruction of intestinal epithelial structures, usually characterized by bloody diarrhea [1]. The severity and duration of colonic mucosal congestion are strongly associated with the risk of cancer. Previous statistics have shown that the incidence of UC is higher in developed countries; however, it is gradually increasing in Asia [2]. Currently, reducing inflammatory symptoms in the gut remains the mainstay of colitis treatment, including the use of 5-aminosalicylic acid (5-ASA)-based drugs, steroids, glucocorticoids, and immunosuppressants. These drugs have shown many side effects and unstable efficacy following long-term medication [3]. Relieving inflammatory symptoms and regenerating damaged intestinal mucosa are still challenges in current treatment. The etiology of UC remains incompletely elucidated.

*Smilax china* L., Smilacaceae (SC), also known as Jingangteng, has been used medicinally in China for over a thousand years [4]. China is rich in plants of SC, with cultivated medicinal SC primarily found in Hubei and Hunan provinces. Modern phytochemical studies reveal its rich composition of bioactive compounds, including flavonoids, polyphenols, and polysaccharides, etc., which contribute to its anti-inflammatory, lipid-lowering, and anticancer properties [5,6]. Jingangteng capsule (JGTC) is made from the supernatant obtained by water extraction and alcohol precipitation using SC as a raw material. JGTC is widely used in the treatment of gynecological inflammations such as pelvic inflammatory disease, and has a high market share due to its significant therapeutic effects and safety [7]. However, the potential of JGTC to suppress inflammation and restore the intestinal barrier in UC has not yet been explored.

Advancements in multi-omics platforms such as metabolomics and 16S rDNA sequencing are deepening our understanding of UC. Metabolomics provides a reliable material foundation for investigating the therapeutic mechanisms of plant-derived medicines and their effective components. Due to the multi-component and multi-target characteristics, traditional Chinese medicine (TCM) is commonly regarded as capable of significantly correcting metabolic dysregulation. With the progress in sequencing technologies, the pivotal role of gut microbiota in the progression and treatment of UC is being increasingly recognized. Correcting dysbiosis offers an effective means to relieve UC [8]. TCM is generally administered orally to treat diseases via the digestive tract. During gastrointestinal metabolism, TCM components can act on the gut microbiota. By reshaping microbial composition and influencing immune response and metabolism, these components help rebuild the intestinal mucosal barrier [9]. Active compounds in TCM can also be structurally remodeled by gut microbial enzymes, affecting their pharmacological and even toxicological activity [10]. This effect may occur through the regulation of intestinal flora, inflammatory factors, modulation of the immune system, and protection of the intestinal mucosa.

The interactions between bioactive compounds and their potential targets can be predicted through network pharmacology. This analysis method further enhances sequencing technologies, aiding research into complex mechanisms of action. Combining network pharmacology with metabolomics not only accelerates compound research but also offers deeper insights into the synergistic effects and potential mechanisms of plant-based medicine components. This integrated approach greatly enhances the safe development of botanical therapies, accelerating innovation in the research and development of natural medicines.

**Figure 1:**
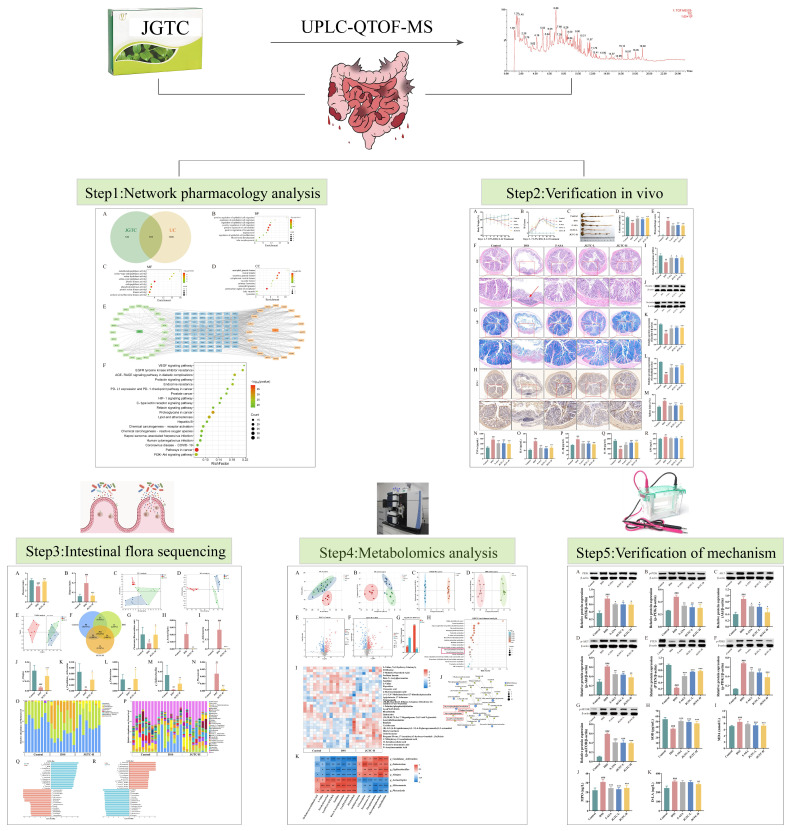
Flow chart of the study. # *p* < 0.05, ## *p* < 0.01, ### *p* < 0.001, vs. the control group; * *p* < 0.05, ** *p* < 0.01, *** *p* < 0.001, vs. the DSS group.

This study aimed to investigate the efficacy and potential mechanisms of JGTC in the treatment of UC by combining the expanded application of the drug with modern pharmacological methods. At present, a combination of network pharmacology, experimental validation, gut microbiota analysis, and metabolomics has emerged as an effective approach for elucidating the mechanisms of action of TCM in complex diseases, including inflammatory conditions. Accordingly, this study employed the integrated approach to explore the effects of JGTC on UC symptoms, gut microbiota dysbiosis, and metabolic disturbances, confirming the drug’s efficacy and systematically identifying key signaling pathways (Figure 1).

## 2. Results

### 2.1. Chemical Components of JGTC

**Figure 2:**
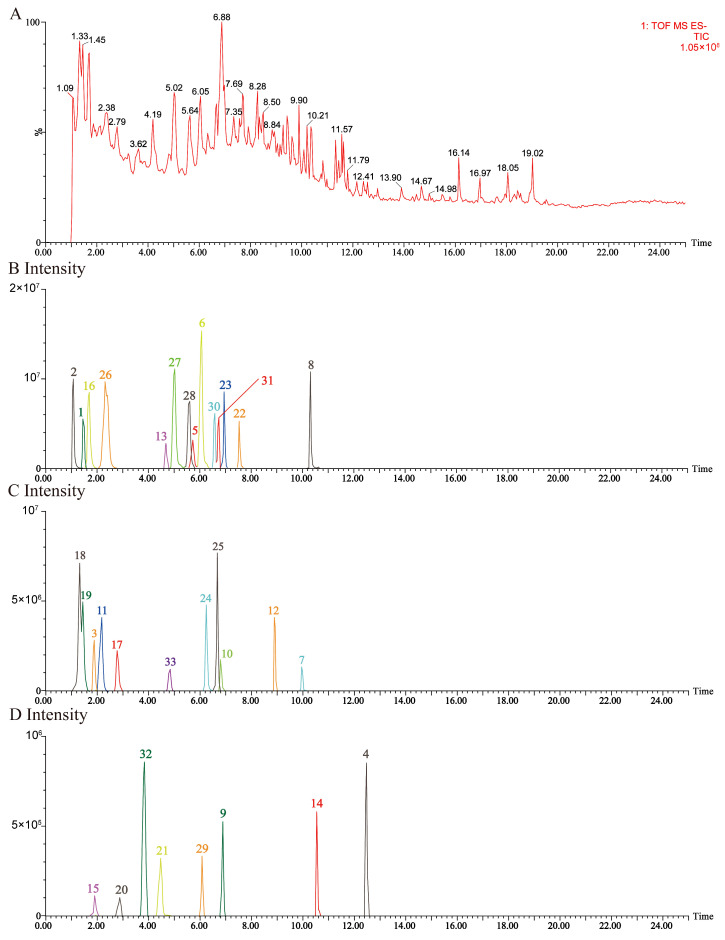
Ion chromatograms of JGTC in negative-ion mode. (**A**) Total ion chromatogram. (**B**–**D**) Extracted ion chromatograms based on UPLC-QTOF-MS/MS profiles. The compound numbers correspond to Table 1.

**Figure 3:**
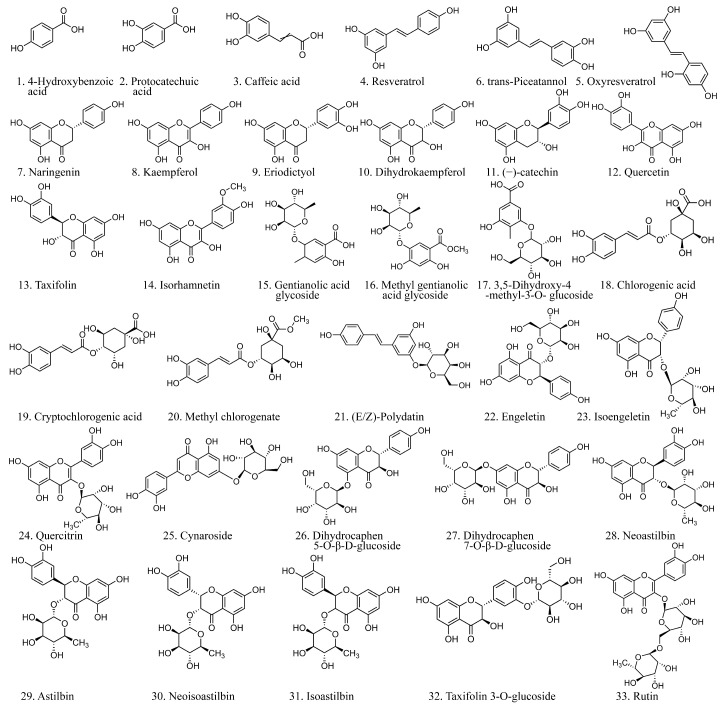
Chemical structure of components identified in JGTC. The compound numbers correspond to Table 1.

**Figure A1:**
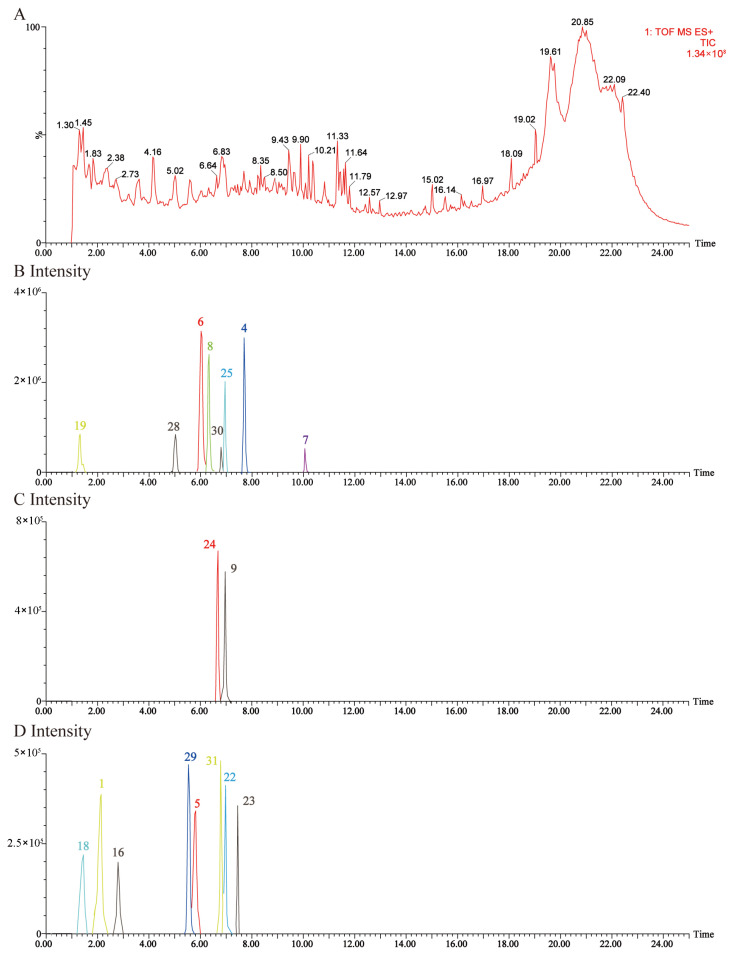
(**A**) Total ion chromatogram of JGTC in positive-ion mode. (**B**–**D**) Extracted ion chromatogram based on UPLC-QTOF-MS/MS profiles in positive-ion mode. The compound numbers correspond to Table 1.

We analyzed the major components of JGTC using UPLC-QTOF-MS/MS, identifying 33 compounds in negative-ion mode (Table 1 and Figure 2). There were 18 compounds identified in positive-ion mode, with details provided in Table A1 and Figure A1. The chemical structure of the 33 compounds is shown in Figure 3.

### 2.2. Network Pharmacology Analysis

**Figure 4:**
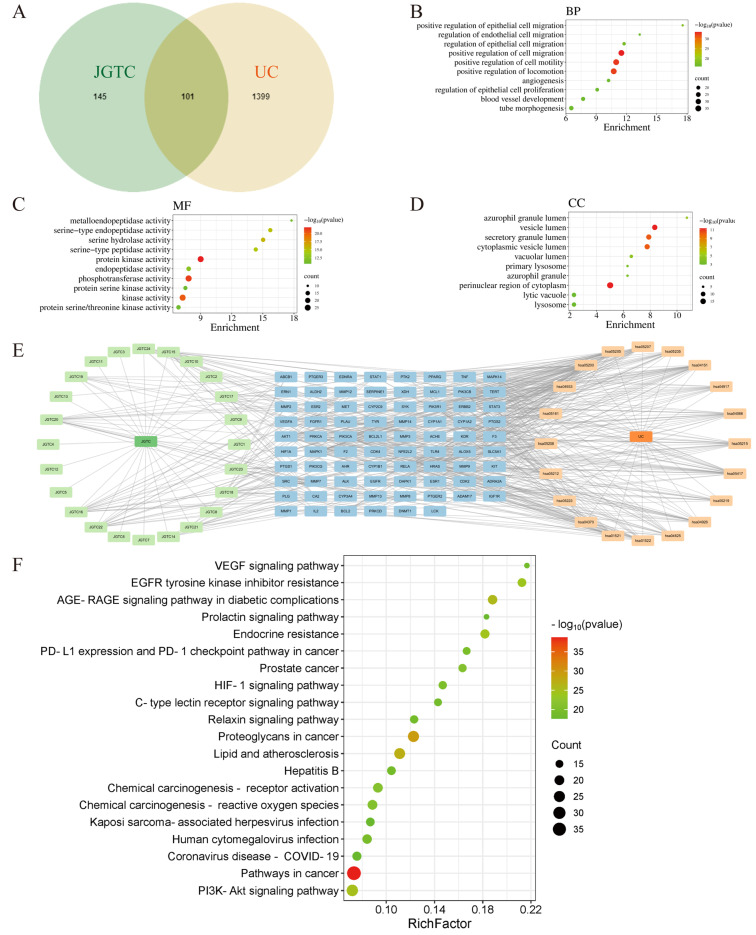
Network pharmacology analysis. (**A**) Venn map related to overlapping genes from bioactive compounds in JGTC and UC–related genes. (**B**–**D**) GO pathway enrichment analysis of the overlapping genes of JGTC in UC. (**E**) JGTC–Component–Target–Pathway–UC visualization network. (**F**) KEGG pathway enrichment analysis of overlapping genes in JGTC for UC.

A total of 33 compounds detected in JGTC were analyzed. Protein targets for 24 of these compounds (Table A2) were identified through PubChem and Swiss Target Prediction databases. A total of 246 target proteins were identified as potent active JGTC compounds, based on the drug target database. Using the disease target database, the top 1500 potential therapeutic targets for UC were predicted and selected. We recognized 101 overlapping targets as potential targets of JGTC for UC treatment (Figure 4A). In the protein–protein interaction (PPI) network, proteins were represented by nodes, while protein interactions were represented by edges. Nodes of different colors and sizes indicate different degree values, with larger and redder colors denoting higher degree values and more critical targets. We got 550 Gene Ontology (GO) items by introducing the key active cross-targets of JGTC and UC into the MetScape platform. The bubble diagram of GO highlighted biological processes: hormone regulation, LPS response, hypoxia adaptation, and inflammation. The major molecular response functions were transcription factor binding, nuclear receptor activity, phosphatase binding, and oxidoreductase activity (Figure 4B–D). The “Drug–Component–Target–Pathway–Disease” network identified 4-hydroxybenzoic acid, caffeic acid, resveratrol, oxyresveratrol, trans-piceatannol, naringenin, kaempferol, eriodictyol, and quercetin as top-degree nodes, indicating their multi-target potential (Figure 4E). The above studies implied that the process of JGTC to improve UC mainly involves the regulation of inflammatory responses and angiogenesis.

The Kyoto Encyclopedia of Genes and Genomes (KEGG) signaling pathway enrichment results showed that the mechanism of action of JGTC on UC may be related to PI3K-Akt, reactive oxygen species, endocrine resistance, HIF-1 signaling pathway, and vascular endothelial growth factor signaling pathways (Figure 4F). Core targets for network pharmacology analysis, such as PIK3CA, PIK3CB, and AKT1, are also key points in the PI3K-Akt pathway. Caffeic acid, resveratrol, naringenin, kaempferol, and quercetin, as the core components of JGTC, have inhibitory effects on the PI3K-Akt (m-TOR) pathway. Oxyresveratrol and trans-piceatannol are similar in structure and action to resveratrol. The results of network pharmacology revealed that PI3K-Akt (m-TOR) is an important pathway for the action of JGTC on UC.

### 2.3. Amelioration of UC Symptoms, Intestinal Barrier, and Inflammation

**Figure 5:**
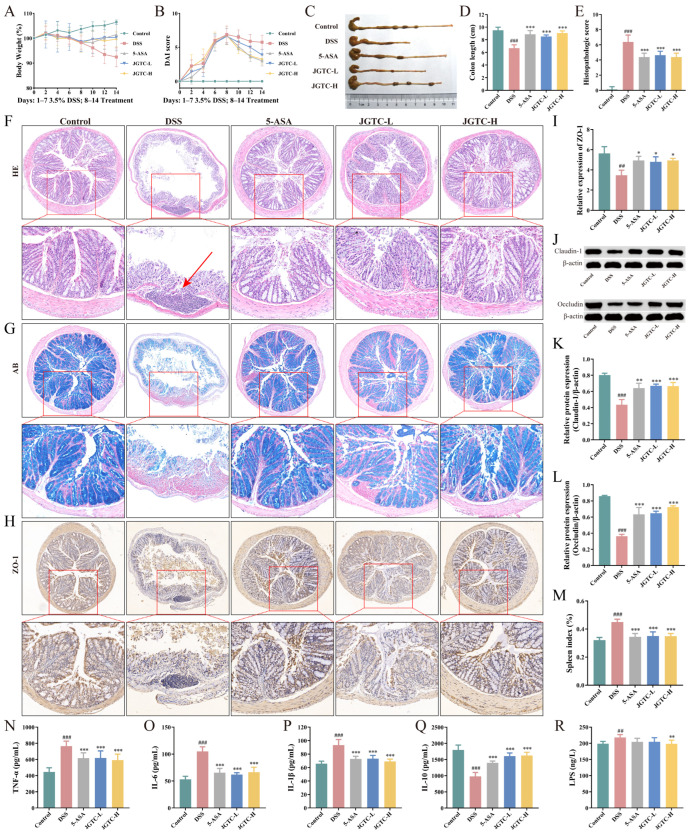
The activity evaluation of JGTC in DSS-induced UC mice. (**A**) Curves of body weight change. (**B**) Curves of DAI changes. (**C**) Colon length after JGTC treatment. (**D**) Statistical results of colon length in each group (*n* = 8). (**E**) Morphological scores of colon sections (*n* = 3). (**F**) Representative images of HE staining. (**G**) Representative images of AB staining. (**H**,**I**) Expression of ZO-1 in the colon (*n* = 3). (**J**–**L**) Expression of Claudin1 and Occludin in the colon (*n* = 3). (**M**) Statistical result of spleen index. (**N**–**R**) Serum levels of TNF-α, IL-6, IL-1β, IL-10, and LPS (*n* = 8). ## *p* < 0.01, ### *p* < 0.001, vs. the control group; * *p* < 0.05, ** *p* < 0.01, *** *p* < 0.001, vs. the DSS group.

A model of 3.5% DSS-induced UC mice was employed to evaluate the protective effect of JGTC. The therapeutic outcomes were presented in Figure 5. It was evident that over the 7 days of DSS administration, the mice exhibited a persistent decline in body weight, serving as a measure of acute colitis severity. Concurrently, the mice displayed marked bloody stools, indicating successful model establishment. Following drug treatment, the weight loss was reversed, showing a noticeable difference compared to the DSS group (Figure 5A). Regarding disease activity index (DAI) scores, the high scores induced by DSS were effectively reduced by both 5-ASA and JGTC, demonstrating excellent recovery effects (Figure 5B). The reduction in colon length presented diminished physiological function and served as a crucial indicator of colitis. In comparison to the control group, the colons of DSS-induced mice were significantly shortened, while those in the treatment groups were closer to the control group (Figure 5C,D). HE staining provided clear, direct observation of the colon’s microscopic tissue morphology. Results of the staining revealed severe pathological alterations in colitis-induced mice, including inflammatory cell infiltration, glandular damage, and reduced mucosal surface area. Post-treatment, colonic pathology improved, characterized by relatively regular glandular arrangement, restored goblet cells, reduced inflammatory cell infiltration, and enhanced colonic tissue integrity (Figure 5E,F). The red arrow in Figure 5F indicates an ulcer in the colon, with loss of crypts, which severely impairs colonic function. Compared to the DSS group, both 5-ASA and JGTC demonstrated significantly reduced histopathological scores. AB staining provided superior visualization of goblet cell status. The stained images revealed severe damage to the mucosal layer in the colon of colitis mice, resulting in a precipitous decline in goblet cell numbers. Both drugs increased goblet cell counts, restoring colonic mucus secretion, which is a critical component of the intestinal barrier (Figure 5G). These findings indicate that both dosages of JGTC exert therapeutic effects against UC. For the majority of data points, the effects of high-dose JGTC were closer to those of 5-ASA, suggesting that clinically equivalent doses of JGTC are effective in alleviating inflammatory symptoms in UC mice.

Damage to the intestinal barrier is one of the manifestations and hallmarks of UC. Tight junction (TJ) proteins form the epithelial cell skeleton, serving as critical factors in maintaining the structural and functional integrity of the intestinal mucosa. In colitis, the reduction in TJ proteins constitutes a major cause of impaired barrier function. Abnormally increased intestinal permeability disrupts the selective passage of substances, allowing harmful agents to exploit this vulnerability. We employed IHC and WB to examine the expression of TJ proteins in the colon. Significant downregulation of ZO-1, Claudin-1, and Occludin was observed in the DSS group colon. Expression levels of these proteins recovered in the three treatment groups, approaching those of the normal control group (Figure 5H–L). This improvement in protein expression demonstrated that JGTC can improve the intestinal barrier.

Abnormal changes in inflammatory mediators profoundly contribute to the onset of inflammation in UC. The hyperactivity of pro-inflammatory factors and the suppression of anti-inflammatory factors lead to uncontrolled intestinal inflammation. The state of intestinal inflammation can be reflected by cytokine expression levels. Compared to the control group, serum levels of TNF-α, IL-6, and IL-1β were elevated in colitis mice. Following administration, these pro-inflammatory factors were markedly suppressed (*p* < 0.001, Figure 5N–P). Conversely, the anti-inflammatory factor IL-10 exhibited enhanced expression following drug administration (*p* < 0.001, Figure 5Q). LPS not only activates inflammatory pathways but also directly compromises the intestinal barrier. Its dysregulation stems from dysbiosis. Following intestinal barrier damage, LPS translocates into the lamina propria, leading to increased pro-inflammatory factors. This further deteriorates the intestinal barrier, creating a vicious cycle in UC. Among the three treatment groups, only the JGTC-H group exhibited significantly lower LPS levels than the DSS group, suggesting a marked inhibitory effect of high-dose JGTC on LPS (*p* < 0.01, Figure 5R). The spleen constitutes a vital component of the immune system, involved in the production, storage, and release of immune cells. The explosive, inflammatory environment severely compromises the spleen, manifesting externally as increased weight, size, and spleen index. Following drug treatment, spleen weight and size, as well as the spleen index, were significantly reduced. Therapeutic efficacy was comparable across the three treatment groups, with minimal difference from the control group (*p* < 0.001, Figure 5M). These findings indicated that JGTC exerts a mitigating effect on UC by restoring the intestinal barrier and restraining inflammation.

### 2.4. Improvement in Intestinal Flora Disorder with JGTC Treatment

**Figure 6:**
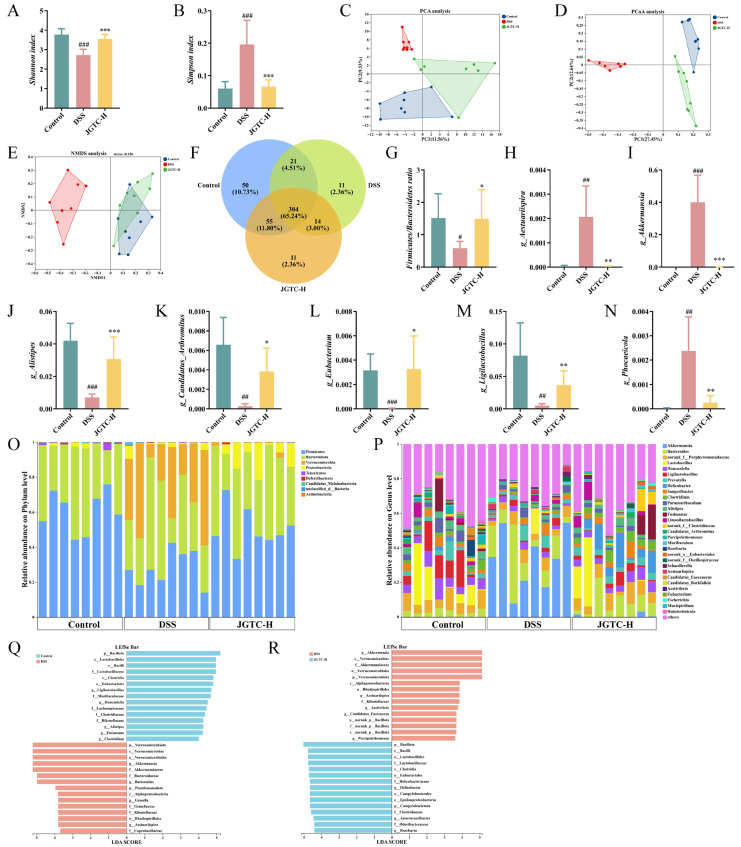
The recovery of disordered gut microbiota after the effect of JGTC. (**A**) Shannon index (*n* = 8). (**B**) Simpson index (*n* = 8). (**C**) Principal component analysis. (**D**) Principal coordinate analysis. (**E**) Non-metric multidimensional scaling. (**F**) Venn diagram of the intestinal microflora. (**G**) Firmicutes/Bacteroidota ratio at the phylum level (*n* = 8). (**H**–**N**) Changes in abundance at the genus level (*n* = 8). (**O**) Differences at the phylum level. (**P**) Differences at the genus level. (**Q**,**R**) Discriminant analysis of multilevel species differences in LEfSe (*n* = 8). # *p* < 0.05, ## *p* < 0.01, ### *p* < 0.001, vs. the control group; * *p* < 0.05, ** *p* < 0.01, *** *p* < 0.001, vs. the DSS group.

We performed 16S rDNA sequencing on fecal samples from control, DSS, and JGTC-H (1.24 g/kg) groups to assess the impact of JGTC on the gut microbiota of UC mice. Judging by the α-diversity exhibited in Shannon and Simpson indices, JGTC restored both the diversity and abundance of the disrupted gut microbiota (Figure 6A,B). Analysis of β-diversity compares groups to explore similarities or differences in overall community structure. In the scatter plots of PCA, PCoA, and NMDS, the control groups and JGTC-H partially overlapped or showed slight separation, indicating similar or closely related community structures. Both groups were distinct from group DSS, suggesting relatively greater differences in community structure (Figure 6C–E). Venn diagrams compared unique and shared operational taxonomic units (OTUs) (defined at 97% similarity) across groups, visualized compositional overlap, and distinct OTU profiles. The control, DSS, and JGTC-H groups contained 50, 11, and 11 unique OTUs, respectively (Figure 6F).

At the phylum level, JGTC increased the relative abundance of *Actinobacteria*, *Deferribacteres,* and *Firmicutes*, which were reduced by DSS, and decreased the relative abundance of *Candidatus_Melainabacteria* and *Verrucomicrobia* (Figure 6O). *Firmicutes* and *Bacteroidota* phyla were dominant species in each group; JGTC restored the sliding *Firmicutes/Bacteroidota* (F/B) ratio (Figure 6G). At the genus level, the relative abundance of *Ligilactobacillus*, *Candidatus_Arthromitus*, *Alistipes,* and Eubacterium was upregulated after treatment, while that of *Akkermansia*, *Aestuariispira*, and *Phocaeicola* was reduced (*p* < 0.05 and *p* < 0.01, Figure H–N, P). Administration of JGTC meaningfully reversed these genus-level differences, and the relative abundance levels of intestinal flora were similar to those in the control group.

**Figure A2:**
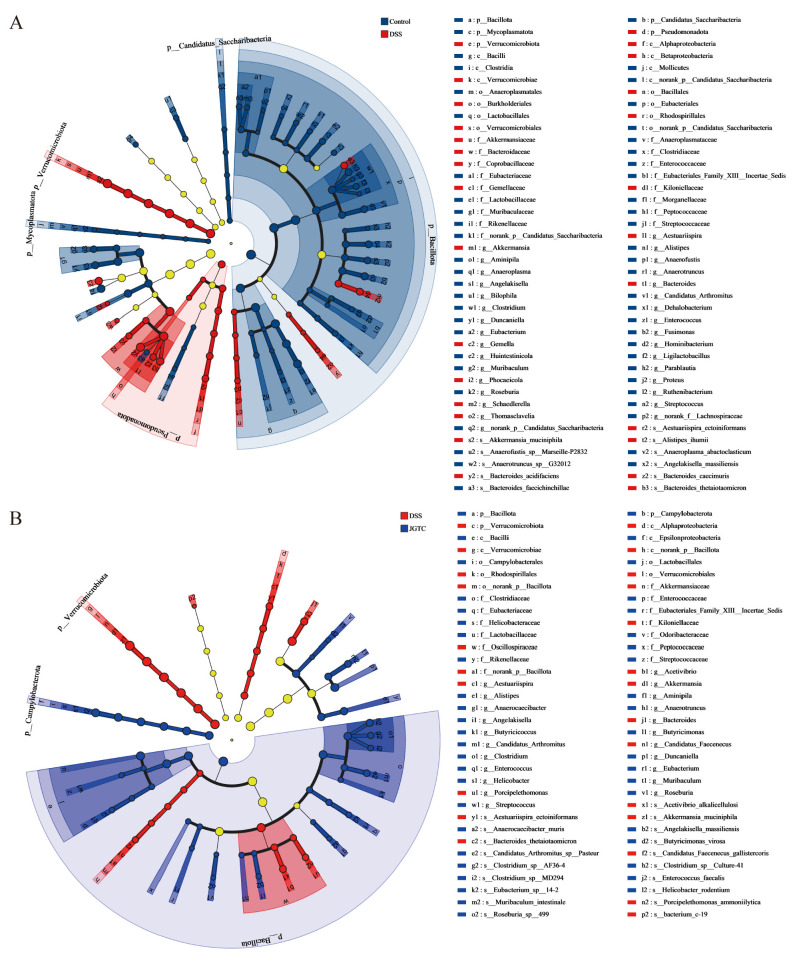
(**A**) LEfSe analysis between the control and DSS groups. (**B**) LEfSe analysis between DSS and JGTC groups.

LEfSe analysis identified taxonomic differences across groups (Figure A2). LDA bar plots highlighted taxa with significant differential abundance (LDA score > 2). Compared to the control group, the *p_Verrucomicrobiota*, *c_Verrucomicrobiae*, *o_Verrucomicrobiales*, *g_Akkermansia,* and *f_Akkermansiaceae* in the DSS group exhibited greater variability (Figure 6Q). When it came to the comparison with the DSS group, *p_Bacillota*, *c_Bacilli*, *o_Lactobacillales*, *f_Lactobacillaceae*, and *c_Clostridia* ranked ahead (Figure 6R). We can observe that the most significant differences in LDA scores were concentrated among a few distinct bacteria. This indicated that they constituted a certain quantitative advantage in the microbial community, and fluctuations in their abundance are likely to exert a substantial influence on the progression of UC.

**Figure 7:**
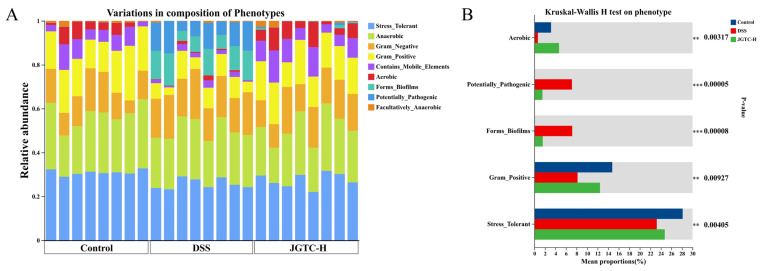
Phenotype prediction by BugBase. (**A**) Variations in Phenotype. (**B**) Intergroup comparison of phenotypic variation (*n* = 8). ** *p* < 0.01, *** *p* < 0.001.

BugBase Phenotype Prediction identifies high-level phenotypes present in control versus treated samples, enabling functional prediction of bacterial data. It directly infers microbial physiological traits from 16S rDNA data, tracking the evolution of microbial functional phenotypes during disease progression. Through phenotype prediction, BugBase links taxonomic shifts to host-microbe interaction mechanisms. Phenotypic alterations were most pronounced in Mobile Element Containing and Biofilm Forming (Figure 7A). There were significant changes in bacterial abundance, with phenotypic features related to five aspects: aerobic, potentially pathogenic, biofilm-forming, Gram-Positive, and stress-tolerant. Following the onset of ulcerative colitis, there was an overall reduction in Gram-positive bacteria, which constitutes one of the core characteristics of dysbiosis. Compared with the normal group, both latent pathogens and biofilm-forming bacteria increased (Figure 7B). These predictions revealed certain mechanisms underlying UC in relation to the gut microbiota.

### 2.5. Improvement of Metabolites in UC Mice Following Administration of JGTC

**Figure 8:**
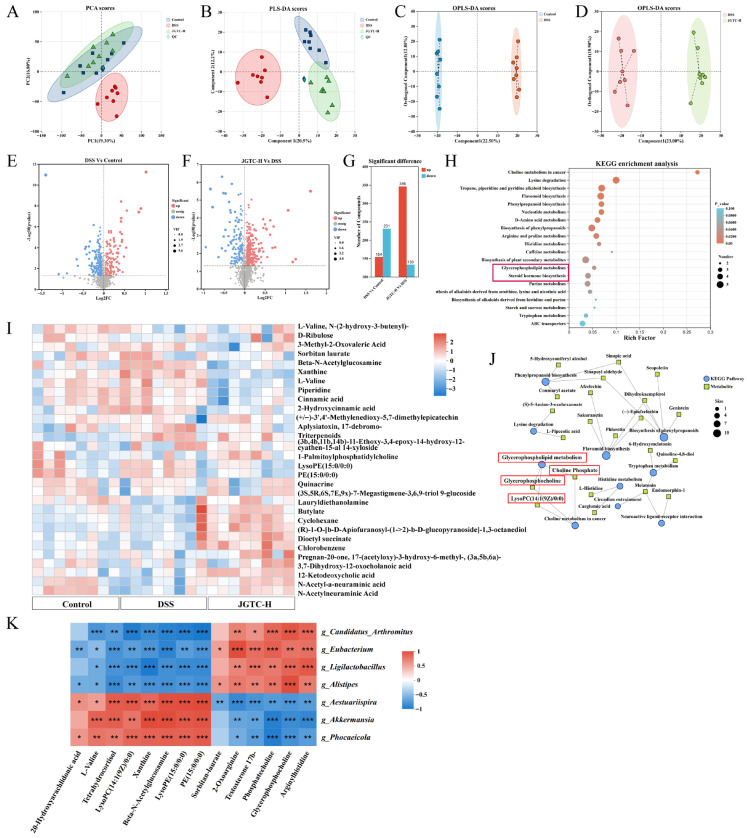
Assessment of fecal metabolites by untargeted metabolomics. (**A**) Scatter plots of PCA scores. (**B**) Scatter plots of PLS-DA scores. (**C**) OPLS-DA score plot between the control and DSS groups. (**D**) OPLS-DA score plot between DSS and JGTC-H groups. (**E**) Volcano plot of differential fecal metabolites in negative-ion mode between the control and DSS groups. (**F**) Volcano plot of differential fecal metabolites in negative-ion mode between DSS and JGTC-H groups. (**G**) Number of differential metabolites. (**H**) KEGG pathway enrichment analysis. (**I**) Heatmap of significantly different metabolites. (**J**) KEGG enrichment analysis network diagram. (**K**) Pearson’s correlation analysis (*n* = 8). * *p* < 0.05, ** *p* < 0.01, *** *p* < 0.001.

Beyond nutrient digestion and absorption, the intestinal tract plays critical roles in metabolic regulation and host–microbiota metabolism. We analyzed the fecal metabolic profiles of mice using untargeted metabolomics, including the control, DSS, and JGTC-H (1.24 g/kg) groups, to explore the alterations of metabolites and metabolic pathways by JGTC treatment. PCA and PLS-DA score plots showed a clear separation between the three groups, with the JGTC-H group samples located between the control and DSS groups (Figure 8A,B). OPLS-DA was employed for further differential metabolite identification. The OPLS-DA score plots of pairs of groups are displayed in Figure 8C,D. The different fecal metabolites in negative-ion mode were presented in volcano plots (Figure 8E,F). Red and blue dots represented metabolites with significant differences in upregulation and downregulation, respectively. The size of the dots indicated the variable importance in projection (VIP) values. Compared to the control group, there were 154 fecal metabolites that rose in UC mice, while 231 decreased. The number of metabolites in the JGTC-H group that increased and decreased was 346 and 133, in comparison with the DSS group (Figure 8G).

Metabolite cluster analysis revealed that the majority of the top thirty differential metabolites in the three groups were categorized as amino acids (AA), glucose, and lipids (Figure 8I). The differentially expressed metabolites (DEMs) were further identified by the thresholds of a *p* < 0.05 for Student’s *t*-test and VIP > 1 for the OPLS-DA model. After screening, 72 and 55 known DEMs were identified in the DSS versus control and JGTC-H versus DSS groups, respectively (Table 2). JGTC reversed the majority of metabolites that were numerously altered in UC. VIP scores were used to display the top-ranked compounds (Figure 8K). Compared with the DSS group, dehydrovomifoliol, linatine, and eugenin increased in the JGTC-H group, while cadaverine and sepiapterin decreased. The reduction in sepiapterin is therapeutic, and supplementation may improve UC.

Relevant metabolic pathways were predicted by KEGG analysis (Figure 8H). Differential metabolite-enriched genes were mainly in AA and lipid metabolism, including arginine, histidine, proline, and lysine metabolism, steroid hormone biosynthesis, and glycerophospholipid metabolism. The relationship between the Top 10 pathways and their associated differential metabolites was illustrated in an enrichment analysis network diagram (Figure 8J). These differential metabolites and the predicted metabolic pathways collectively suggested that JGTC may exert effects on colitis primarily through modulation of AA and lipid metabolism.

Furthermore, Pearson correlation analysis was used to discern associations between gut bacteria at the genus level and differential metabolites. The results revealed that phosphatecholine and glycerophosphocholine were negatively correlated with *Aestuariispira*, *Akkermansia*, and *Phocaeicola*, while demonstrating positive correlations with *Candidatus_Arthromitus*, *Eubacterium*, *Ligilactobacillus,* and *Alistipes*. Most of the remaining differential metabolites were positively correlated with *Aestuariispira*, *Akkermansia*, and *Phocaeicola*, while negatively correlated with other bacteria (Figure 8K).

### 2.6. The Effect of JGTC on Inflammation via the PI3K-AKT-mTOR Pathway

**Figure 9:**
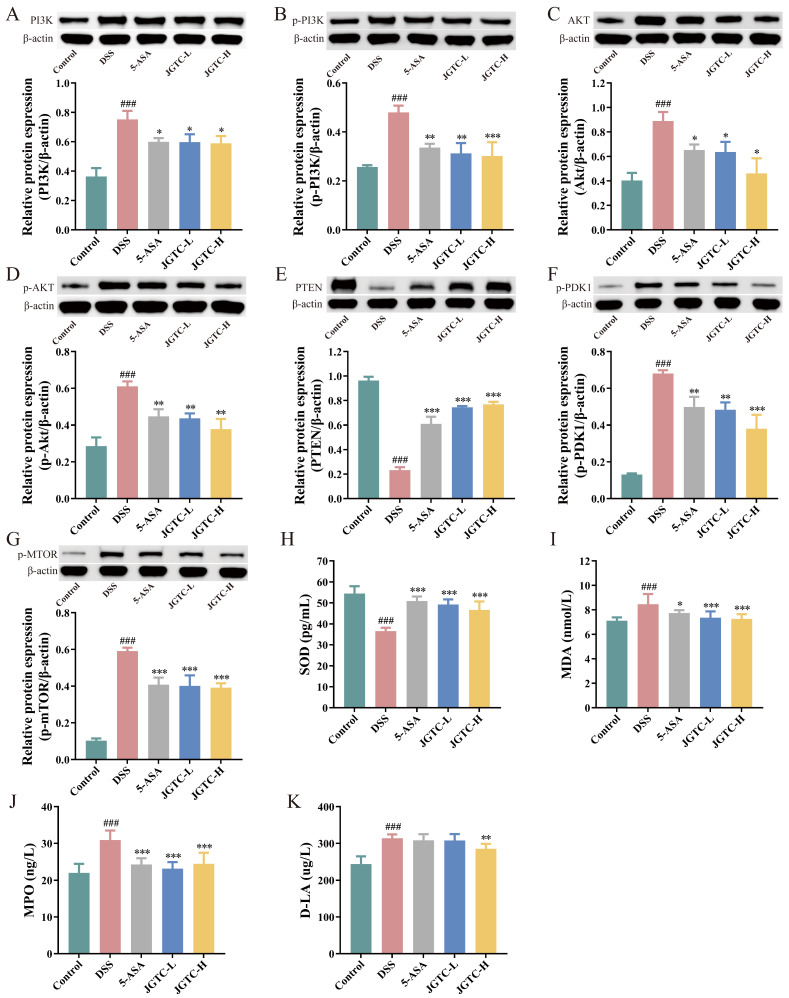
The inhibition of the PI3K-AKT-mTOR pathway by JGTC against UC. (**A**–**G**) The expression of PI3K, p-PI3K, Akt, p-Akt, PTEN, p-PDK1 and p-mTOR (*n* = 3). (**H**–**K**) Serum levels of oxidative stress factor SOD, MDA, MPO, and D-LA (*n* = 8). ### *p* < 0.001, vs. the control group; * *p* < 0.05, ** *p* < 0.01, *** *p* < 0.001, vs. the DSS group.

Many kinds of AA, including arginine, lysine, and proline, share the Rag-Ragulator-lysosomal system as the core platform for mTORC1 activation. Multiple substances involved in lipid metabolism, such as steroid hormone metabolism and glycerophospholipid metabolism, form a complex network of interactions with the PI3K-AKT-mTOR pathway. These pathways and their metabolites form a complex network of interactions with the pathway. Combined with the signaling pathways enriched by network pharmacology, we speculated that JGTC may alleviate UC by modulating key enzymes and associated oxidative stress factors of the PI3K-AKT-mTOR pathway. The signaling pathway plays a central role in the cell. PI3K can activate amplification of the Akt signaling cascade [11]. PDK1 assists in the first phosphorylation activation of AKT, and PTEN reverses this phosphorylation [12]. The mammalian mechanistic target of rapamycin (mTOR) is a key downstream target of the PI3K-Akt signaling pathway [13]. The abnormal damage to intestinal epithelial cells caused by oxidative stress leads to destruction of mucosal barrier function [14]. Inhibiting the PI3K-AKT-mTOR pathway reduced oxidative stress and inflammation in colitis models. Both doses of JGTC significantly downregulated the expression of PI3K and AKT in the colon of UC mice, including in the phosphorylated and non-phosphorylated states. That suppressed the key regulators of the pathway (Figure 9A–D). The expression of phosphorylated PDK1 and mTOR was attenuated after treatment, while that of PTEN was enhanced (Figure 9E–G).

**Figure 10:**
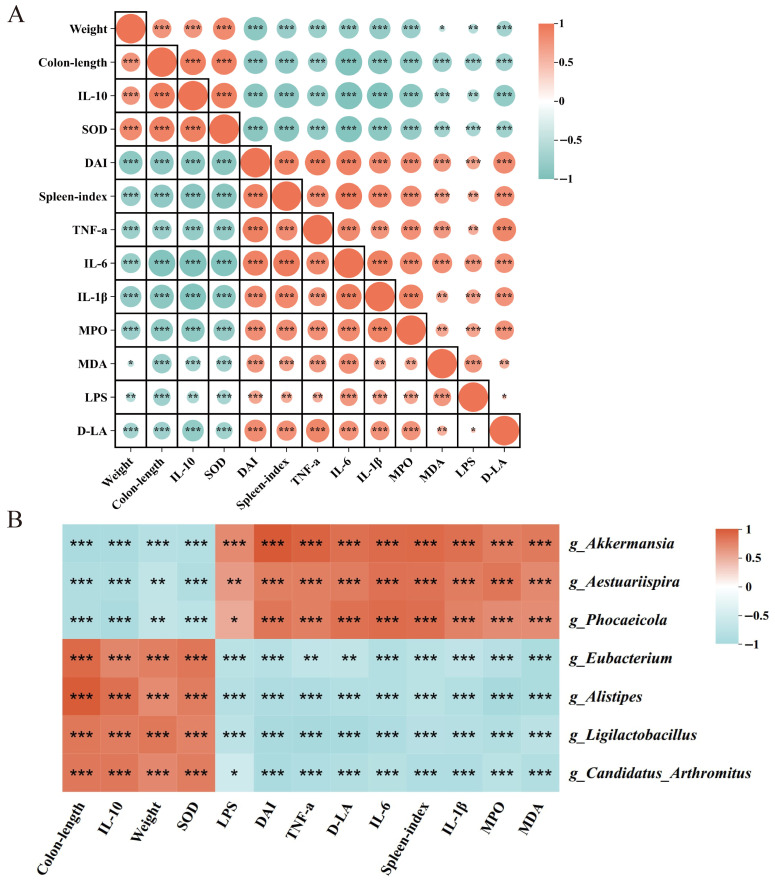
Pearson’s correlation analysis. (**A**) Analysis among daily indices, inflammation, and oxidative stressors (*n* = 8). (**B**) Analysis of genus-level differential intestinal flora and key physiology factors. * *p* < 0.05, ** *p* < 0.01, *** *p* < 0.001.

As a core antioxidant enzyme, changes in SOD activity directly influence reactive oxygen species (ROS) levels. In UC, increased intestinal epithelial permeability allows D-lactate (D-LA) to enter the bloodstream through the compromised barrier from the intestinal lumen. Consequently, serum D-LA levels serve as a marker reflecting barrier damage; its accumulation induces ROS production, which in turn activates the PI3K-AKT-mTOR pathway by oxidizing negative regulators such as PTEN. MPO, acting as an executor of neutrophil activation, directly injures tissues by generating oxidants and may activate this pathway via ROS. MDA is the end product of lipid peroxidation, reflecting not only the extent of oxidative damage but also indirectly influencing the PI3K-AKT-mTOR pathway. In terms of oxidative stress, the ELISA results demonstrated that JGTC restored SOD levels and decreased MDA and MPO levels (*p* < 0.05 and *p* < 0.001, Figure 9H–J). D-LA levels were effectively declined in the JGTC-H group (*p* < 0.01, Figure 10 K). The regulation of JGTC on key factors in the PI3K-AKT-mTOR pathway and associated oxidative stress factors, particularly its beneficial effects at high doses, indicated that it alleviates inflammatory responses in UC by inhibiting this pathway.

Pearson correlation analysis revealed that weight, colon length, levels of IL-10 and SOD were negatively related to DAI scores, spleen index, and the levels of remaining inflammation and oxidative stress factors (Figure 10A). Changes in intestinal flora, especially at the genus level, were strongly associated with pathological deterioration in UC mice (Figure 10B). *Akkermansia*, *Aestuariispira*, and *Phocaeicola*, which were more abundant in the DSS group, were negatively related to weight, colon length, levels of IL-10, and SOD. DAI scores, spleen index, and the levels of remaining inflammation and oxidative stress factors were positively related to these genera. Other related results were contrary to the aforementioned findings. The levels of differential genera were strongly associated with a range of pathologies in colitis, particularly numerous factors related to inflammation and oxidative stress. It can be inferred that the disease-related manifestations are strongly associated with the alterations and function of intestinal flora during UC.

## 3. Discussion

JGTC, which is made from the supernatant obtained by aqueous extraction and alcohol precipitation of SC, mainly retains flavonoids and polyphenols as the active medicinal components and is primarily characterized by its anti-inflammatory properties [15]. Recent studies have shown that JGTC has a hypoglycemic and lipid-lowering effect [16]. Based on these pharmacological activities, we hypothesized that JGTC has the potential to improve UC. We identified the chemical components of JGTC as the basis for screening natural anti-UC active ingredients. After treatment, model mice regained body weight, showed decreased DAI scores, and restored pathological damage to the colon. The severely damaged intestinal barrier was improved.

The flavonoids and polyphenolic compounds detected in JGTC exert synergistic effects on UC via multiple targets and pathways. Quercetin has been shown to inhibit the inflammatory response and promote mucosal healing by blocking the pathway through the prevention of PI3K and AKT phosphorylation [17]. Flavonoids and polyphenolic compounds are renowned for their potent antioxidant capacity. They combat oxidative stress by multiple pathways and protect mitochondrial function. Many of them are also powerful free radical scavengers. The phenolic hydroxyl groups enable them to react with free radicals, thereby terminating free radical chain reactions. Additionally, they can enhance the activity of endogenous antioxidant enzymes such as SOD. The compounds also help restore immune homeostasis by modulating the function of various immune cells. As most flavonoids and polyphenolic compounds are poorly absorbed by the small intestine, they reach the colon, interacting with the gut microbiota. They improve the intestinal barrier by regulating the composition and function of the microbiota through increasing the abundance of beneficial bacteria and promoting the production of short-chain fatty acids.

With the study of intestinal flora, its complex relationship with diseases is becoming apparent. One of the primary indicators of gut health is the diversity and abundance of gut flora [18]. A characteristic feature of UC is an abnormal ratio of *Firmicutes/Bacteroidetes* abundance, and the ratio was consistently low in model mice [19,20], but the act of the JGTC-H group was closer to the control group. While the abundance of *g_Akkermansia* decreased following treatment in our study, its role in colitis remains debated [21]. Some research suggests that it is important in the development of UC, which may be related to the genotype of the host, the intestinal micro-ecological environment, and especially the species and abundance of the causative organisms [22,23]. Significant changes in intestinal flora further impact the course of intestinal inflammation.

The PI3K-AKT-mTOR signaling pathway plays a central role in the regulation of UC pathogenesis [24]. Many studies have confirmed significantly elevated expression levels of p-PI3K, p-AKT, p-mTOR, and p-mTOR in active colonic tissue [25]. AKT activates the IκB kinase (IKK) complex through phosphorylation, promoting the ubiquitination and degradation of IκBα, thereby initiating the transcription of pro-inflammatory genes such as TNF-α, IL-1β, and IL-6 [26]. Corresponding to the upregulation of pro-inflammatory factors, excessive activation of the PI3K-AKT-mTOR pathway is accompanied by suppression of key anti-inflammatory cytokine expression. In effector T cells and macrophages, pathway activation promotes inflammatory responses. Conversely, in regulatory T cells (Tregs), excessive activation leads to suppressed Foxp3 expression and functional exhaustion, reducing IL-10 production. The pathway enhances glycolysis and inflammatory cell metabolic reprogramming by promoting HIF-1α translation via mTORC1 [27]. It regulates mRNA stability, prolonging the half-life of inflammatory mediators. Persistent pathway overactivation induces intestinal epithelial cell cycle disruption and increased apoptosis. The pathway regulates TJs by restraining Occludin and ZO-1 expression. It promotes Occludin endocytosis and degradation via mTORC1–S6K1 axis-mediated phosphorylation. Through the ROCK-MLCK pathway, it increases myosin light chain phosphorylation, contracting the cytoskeleton and widening intercellular gaps.

AA possesses high nutritional value and is essential for intestinal growth and the maintenance of mucosal integrity and barrier function [28]. Due to the impaired digestive function of colitis patients, nutrient absorption is compromised, potentially leading to malnutrition. AA, as an adjunctive nutritional therapy, may help to maintain intestinal integrity and reduce inflammation, oxidative stress, and intestinal cell death caused by UC [29]. Arginine is one of the most critical endogenous AA activators of mTORC1 [30]. Within the pathological context of ulcerative colitis, elevated levels of arginine and lysine sustainably activate mTORC1 in intestinal epithelial cells and immune cells. This process drives anabolic pathways, including cell proliferation, protein synthesis, and lipid synthesis. Concurrently, it suppresses catabolic processes such as autophagy, forming a pathological positive feedback loop between metabolism and signaling [31]. Histidine treatment of Caco-2 cells inhibited the secretion of pro-inflammatory factors from intestinal epithelial cells at the transcriptional level [32]. The locally elevated lysine environment in the intestine drives Th1/Th17 response dominance by activating mTORC1. This results in suppressed Treg function, compromised immune tolerance, and exacerbated tissue damage. The proline–mTOR axis exerts multidimensional regulatory effects on intestinal epithelial cell function. Results of metabolic KEGG pathway enrichment and differential metabolite analysis showed that JGTC affected UC by adjusting AA.

Lipids are energy sources, hormones, signaling regulators, and the main building materials of cell membranes. Multiple studies have reported impaired lipid metabolism in colitis patients [33,34]. Lipid metabolism disorders can lead to the abnormal production and secretion of inflammatory mediators, which affect lipid homeostasis in terms of synthesis, structure, and redox processes, subsequently inducing the production of inflammatory mediators in organs such as the intestinal tract [35]. Lipid metabolites may also influence PI3K-AKT-mTOR activity. Free testosterone within the steroid hormone biosynthesis pathway mediates genomic and non-genomic signaling via the androgen receptor (AR), affecting multiple cellular functions. The PI3K-AKT-mTOR pathway represents a key downstream effector of AR signaling. The pathway serves as a key mediator of the non-genomic effects of glucocorticoids, the first-line therapeutic agents for UC [36]. Tetrahydrocortisol levels reflect overall cortisol secretion and metabolic status. Research indicates that palmitic acid may act as a signaling enhancer for adipokines such as adiponectin, promoting the activation of the PI3K-AKT pathway [37]. Choline phosphate (ChoP) is a core intermediate in glycerophospholipid metabolism, generated by choline phosphorylation catalyzed by choline kinase, constituting the rate-limiting step in phosphatidylcholine (PC) synthesis [38]. Although ChoP is not a direct substrate for PI3K, choline metabolism exhibits profound connections with PI3K signaling [39]. PI3K family enzymes are essentially lipid kinases, with Class I members catalyzing the conversion of PIP_2_ to PIP_3_. The polar head groups of both PIP_2_ and PIP_3_ share structural similarities with PC, containing phosphate groups and nitrogenous bases [40]. PA produced by PC cleavage serves as a key activator of the PI3K-AKT-mTOR signaling pathway [41]. It has been found that the activation of IL-6 by PA in macrophages activates PI3K. PA also activates and targets mTOR in mammals [42]. Choline metabolites such as PC, GPC, and LysoPC constitute major components of the cell membrane, and their equilibrium is crucial for maintaining membrane integrity and signaling transduction efficiency. Oxidative stress disturbs intestinal flora and damages intestinal mucosal epithelial cells, leading to the destruction of the mucosal barrier and the immune system [43]. Inhibition of the PI3K-AKT-mTOR pathway can reduce oxidative stress by modulating the secretion of inflammatory cytokines, regulating the expression of antioxidant enzymes, and activating autophagy [44].

*Eubacterium* and *Candidatus_Arthromitus* participate in the metabolism of steroids and bile acids within the gut [45]. Microbiota metabolic activity may indirectly influence the PI3K-AKT-mTOR signaling pathway in intestinal epithelial and immune cells by altering the concentration of bioactive steroids in the gut. *Alistipes* possesses the capacity to produce short-chain fatty acids such as succinate and acetate, while metabolizing bile acids and amino acids to generate bioactive molecules. *Ligilactobacillus* is capable of producing butyrate and propionate [46]. Butyrate, a primary metabolic product of dietary fiber fermentation by gut microbiota, inhibits mTORC1 activity and promotes autophagy [47]. It also suppresses excessive Th17 differentiation, facilitates Treg induction, and restores Th17/Treg balance. Propionate and acetate exert differential regulation on the PI3K-AKT-mTOR pathway via receptors such as GPR41/43 [48]. *Ligilactobacillus* is a probiotic genus recently demonstrated to possess significant anti-UC effects. A study published in 2025 indicates that *Ligilactobacillus plantarum 6C* (L6C), belonging to the *Ligilactobacillus* genus, can inhibit the PI3K-AKT signaling pathway and alleviate DSS-induced colitis symptoms. Transcriptomic analysis revealed that L6C intervention significantly upregulated expression of the *Lama*2 and *Lamb*2 genes. These genes encode the laminin α-2 and β-2 subunits, crucial components of the basement membrane, whose increased expression aids in repairing the intestinal epithelial barrier. Metabolomics further revealed that L6C exerts effects via pathways including mTOR, modulating the spectrum of gut microbiota metabolites. Lipid metabolism pathways such as steroid hormone metabolism and glycerophospholipid metabolism, alongside bacterial genera including Lactobacillus, form deep functional coupling with the PI3K-AKT-mTOR pathway in UC. This coupling governs membrane structural remodeling, inflammation-metabolite feedback loops, and energy redistribution.

Network pharmacology allows for the simultaneous prediction of active signaling pathways by integrating gene expression and protein interaction data. In our study, the co-analysis of metabolomics and network pharmacology focused on the PI3K-AKT-mTOR pathway. Therefore, we examined the expression of the key factors of the pathway, PI3K, Akt, PDK1, PTEN, and mTOR, and the levels of the related oxidative stress factors SOD, MDA, MPO, and D-LA. The results showed that JGTC improved UC by affecting factors in the pathway.

Our preliminary demonstration of the therapeutic potential of JGTC in diseases related to intestinal flora and metabolic disorders, such as UC, is highly innovative and expands the field of JGTC for the treatment of inflammatory diseases. To the best of our knowledge, the present work identified, for the first time, that JGTC ameliorates UC by modulating the intestinal microbiota and the PI3K-AKT-mTOR signaling pathway; this provides some scientific basis for expanding the clinical application and secondary development of JGTC. The practical implication of this study is the identification of novel functions of this clinical drug, including substantial improvements in various UC disease indicators and promising research potential.

However, it remains unknown whether the gut microbiota also regulates UC via the PI3K-AKT-mTOR pathway, and the specific mechanisms involved are unclear. Metabolomics findings indicated that AA metabolism, particularly that of arginine and proline, has a significant impact on UC. These aspects require further elucidation. In future studies, we will investigate the mechanisms underlying the close association between the PI3K-AKT-mTOR pathway and the gut microbiota. Concurrently, we will further explore the ability of JGTC to alter the gut microbiota and AA metabolism in a UC model.

## 4. Materials and Methods

### 4.1. Chemicals and Reagents

Quercitrin (522-12-3), caffeic acid (331-39-5), engeletin (572-31-6), and rutin (153-18-4) were purchased from Chengdu Push Bio-technology Co., Ltd. (Chengdu, China). JGTC (20221226), DSS (J0627C), mesalamine (5-aminosalicylic acid, 5-ASA, DR0677) were supplied by Hubei Furen Pharmaceutical Co., Ltd. (Xianning, China), Dalian Meilun Biotechnology Co., Ltd. (Dalian, China), and Huazhong Haiwei Gene Technology Co., Ltd. (Beijing, China). Alcian Blue stain kit (G1563) and DAB kit (DA1010) were obtained from Beijing Solarbio Science & Technology Co., Ltd. (Beijing, China). Immunohistochemistry kit and antibody ZO-1(21773-1-AP) were supplied by Beijing Zhong Shan-Golden Bridge Biological Technology Co., Ltd. (Beijing, China) and ProteinTech Group (Wuhan, China). ELISA kits for interleukin (IL)-1β (A105903), IL-6 (A105582), IL-10 (A105911), TNF-α (A104732), LPS (A106003), SOD (A105533), MDA (A105554), MPO (A104994), and D-LA (A01250) were bought from Shanghai Fusheng Industrial Co., Ltd. (Shanghai, China). Antibodies Claudin1 (A21971) and Occludin (A25320) were purchased from Wuhan Aibo Teke Biological Technology Co., Ltd. (Wuhan, China). Antibodies PI3K (4257T), p-PI3K (4228T), AKT (4691T), p-AKT (4060T), p-PDK1 (3438T), PTEN (9559T), p-mTOR (5536T) were supplied by Cell Signaling Technology, Inc. (Danvers, MA, USA).

### 4.2. UPLC-QTOF-MS/MS Analysis of JGTC Sample Solution

Capsule contents (20 mg) were dissolved in 10 mL of methanol and sonicated for 30 min. The supernatant was filtered through a 0.22 µm membrane to obtain the sample solution. The mobile phase consisted of 0.1% formic acid in water (A) and acetonitrile (B), delivered at 0.4 mL/min. Gradient elution proceeded as follows: 0–4 min (12–15% B), 4–21 min (15–70% B), and 21–25 min (70–12% B). Mass spectrometric conditions were consistent with those described previously [16].

### 4.3. Functional Annotation, Network Construction, and Analysis

The protein targets of compounds detected in JGTC were identified through Pubchem (accessed on 18 January 2025) and Swiss Target Prediction databases (accessed on 19 January 2025). Employing ulcerative colitis as the keyword, relevant targets were obtained from the GeneCards (accessed on 20 January 2025), OMIM (accessed on 20 January 2025), and TTD (accessed on 21 January 2025) databases. The intersection of the component and disease-related targets was determined using Venny 2.1.0. These intersecting targets were subsequently input into the STRING database for PPI analysis. Cytoscape 3.9.1, sorted according to the degree value of the network nodes, was further utilized to construct the core target PPI network. The cross-metrics of JGTC and UC were entered into the Metscape platform for enrichment analysis. The meta-database was used for GO and KEGG pathways. The top 10 enriched GO terms included cellular component (CC), molecular function (MF), and biological process (BP) categories. Compounds, related targets, pathways, along with KEGG enrichment analysis, were imported into Cytoscape to visualize the Drug–Component–Target–Pathway–Disease network.

### 4.4. Animal Treatments

Male BALB/c mice (6 weeks old) were provided by Three Gorges University (Hubei, China) and housed in the Experimental Animal Center of Hubei University of Traditional Chinese Medicine (HUCMS) with a light/dark cycle of 12/12 h and free access to food and water. All experimental processes were approved by the Animal Ethics Committee of Hubei University of Traditional Chinese Medicine (Approval No. HUCMS00303454). After one week’s acclimatization, 10 of the 80 mice were randomly assigned to the control group (normal), and the others were administered 3.5% DSS solution for 7 d, until the establishment of the UC model. The sulfate groups of DSS directly interact with the colonic epithelial surface and the mucus layer, eliciting a model that closely recapitulates the clinical and histological features of human UC. DAI, including weight loss, stool consistency, and hematologic disorders, was recorded daily using the method outlined in Table A3. Using the random number table method, the model mice (DAI ≥ 5) were randomly divided into four groups (*n* = 10/group): the DSS group, low-dose JGTC (0.62 g/kg; JGTC-L), high-dose JGTC (1.24 g/kg, equivalent to clinical dose; JGTC-H), and 5-ASA (0.2 g/kg). The drugs were administered orally once daily for 7 d to investigate the effect on UC. The DSS and control groups received equivalent volumes of saline orally once daily for 7 d. Fecal samples were collected on day 7. The next day, the mice were anesthetized for blood collection, followed by euthanasia. The colon and spleen were then collected.

### 4.5. HE and AB Staining

Paraffin embedding was performed after fixing the colon tissues with paraformaldehyde. A 4 μm thick specimen was taken and deparaffinized with xylene and ethanol. After rinsing with water, hematoxylin and eosin (HE) and Alcian blue (AB) staining were performed according to standard methods. HE staining: specimens were immersed in hematoxylin for 5 min, stained with eosin dye for 5 min, dehydrated in ethanol, and finally washed with xylene. AB was stained according to the manufacturer’s instructions and dehydrated using the same method as that for HE staining. The HE-stained colonic tissue was evaluated according to the established protocol (Table A4), and a comprehensive histological score was calculated based on epithelial morphology and the degree of inflammatory infiltration.

### 4.6. Protein Extraction and Western Blotting

Proteins were extracted from the mouse colon tissues using the lysis buffer. Protein quantification was performed, separated by SDS-PAGE gel electrophoresis, and transferred onto a PVDF membrane. After blocking, the primary antibody was incubated at 4 °C overnight. The secondary antibody was then incubated at room temperature for one hour. Protein bands were detected, and analysis was performed using ImageJ (1.46 r).

### 4.7. Immunohistochemistry (IHC)

IHC was used to detect the expression of ZO-1 in colon tissue. Paraffin-embedded colon sections were first deparaffinized, followed by antigen retrieval using citric acid. To minimize non-specific binding, the tissue slides underwent permeabilization and blocking steps. After overnight incubation at 4 °C with primary antibodies, the slides were washed and treated with the corresponding secondary antibodies. HRP-labeled secondary antibodies were used in the IHC experiments, followed by DAB staining and Mayer hematoxylin staining. The fixed specimens were observed and photographed under a microscope.

### 4.8. Enzyme-Linked Immunosorbent Assay (ELISA)

Serum samples from each experimental group were collected for the detection of cytokine levels. The assays for IL-6, IL-10, IL-1β, TNF-α, LPS, SOD, MDA, MPO, and D-LA were conducted according to the protocols provided by the ELISA kit manufacturer (Fusheng, Shanghai, China). Then the optical density of the supernatant was measured at a wavelength of 450 nanometers.

### 4.9. Fecal 16S rDNA Analysis

Microbial community genomic DNA was extracted from mouse fecal samples. The DNA extract was checked on 1% agarose gel, and DNA concentration and purity were determined with a NanoDrop2000 spectrophotometer (Thermo Scientific, Waltham, MA, USA). PCR amplification of the full-length 16S rDNA was performed using the extracted DNA as a template and primers with barcodes. In the control, DSS, and JGTC-H groups, three replicates of each sample were performed, and the purified products were detected and quantified using Qubit 4.0. DNA libraries were constructed and sequenced. We used data platforms for the bioinformatic analysis of gut microbiota and Pearson correlation (http://cloud.majorbio.com, www.chiplot.online/).

### 4.10. Metabolomic Analysis of Fecal Samples

Metabolites were extracted from mouse feces. After sample placement and centrifugation, we obtained the supernatant for LC-MS/MS analysis, which was depicted previously [49]. Progenesis QI software (v3.0, Waters, Milford, MLD, USA) and databases (HMDB, Metlin, and Majorbio) were utilized to process the data.

### 4.11. Statistical Analysis

GraphPad Prism 8.0 software was applied for conducting statistical analysis with a significance threshold set at *p* < 0.05. The data were tested for normality and lognormality using the Shapiro–Wilk test. If the data followed a normal distribution, an ordinary one-way ANOVA was performed. For data that did not follow a normal distribution, a Brown-Forsythe and Welch ANOVA test was performed.

## 5. Conclusions

In this study, we investigated the mitigating effect of JGTC on intestinal inflammation in UC mice, conducted a study on the gut microbiota, analyzed the composition of JGTC, and carried out network pharmacological analyses, which were combined with metabolomics results to predict the mechanism of action and experimental validation. Our results demonstrated that the primary active constituents of JGTC are flavonoids and polyphenols. The drug was capable of improving common symptoms in UC mice, such as weight loss, elevated DAI scores, and colonic shortening; repairing damaged intestinal mucosa and barrier function; modulating abnormal inflammatory responses; and restoring disrupted metabolism and gut microbiota. JGTC may exert anti-inflammatory effects by inhibiting key factors in the PI3K-AKT-mTOR pathway and associated oxidative stress factors. These findings suggest the potential of JGTC for the treatment of UC and elucidate the prospects for the secondary development of JGTC for clinical applications.

## Figures and Tables

**Table 1:** Data from UPLC-QTOF-MS/MS on the major JGTC components in negative-ion mode.

Peak No.	Identification	RT [min]	Chemical Formula	Neutral Mass	Calculated[M-H]-(*m*/*z*)	Observed[M-H]-(*m*/*z*)	Error (ppm)	MS/MS
1	4-Hydroxybenzoic acid	1.55	C_7_H_6_O_3_	138.0317	137.0239	137.0220	−13.87	92.9269, 109.0275
2	Protocatechuic acid	1.09	C_7_H_6_O_4_	154.0266	153.0188	153.0195	4.57	109.0275, 153.0170
3	Caffeic acid *	1.95	C_9_H_8_O_4_	180.0423	179.0344	179.0356	6.70	91.0553, 117.0331, 135.0446
4	Resveratrol	12.50	C_14_H_12_O_3_	228.0786	227.0708	227.0723	6.61	185.0249, 164.0081
5	Oxyresveratrol	5.71	C_14_H_12_O_4_	244.0736	243.0657	243.0656	−0.41	201.0181, 174.0055, 130.0424
6	trans-Piceatannol	6.05	C_14_H_12_O_4_	244.0736	243.0657	243.0656	−0.41	159.0454, 129.9747
7	Naringenin	10.05	C_15_H_12_O_5_	272.0685	271.0606	271.0596	−3.69	187.0414, 151.0049, 107.0142
8	Kaempferol	10.36	C_15_H_10_O_6_	286.0477	285.0399	285.0406	2.46	218.0157, 151.0024
9	Eriodictyol	7.04	C_15_H_12_O_6_	288.0634	287.0556	287.0561	17.77	259.0293, 180.0041
10	Dihydrokaempferol	6.83	C_15_H_12_O_6_	288.0634	287.0510	287.0561	1.74	259.0227, 243.0304, 201.0181
11	(−)-catechin	2.14	C_15_H_14_O_6_	290.0790	289.0712	289.0718	2.08	245.0809, 227.0723, 205.0503, 203.0701, 135.0446, 123.0429, 109.0275
12	Quercetin	8.93	C_15_H_10_O_7_	302.0427	301.0348	301.0335	−4.32	107.0142, 121.0297, 151.0024
13	Taxifolin	4.78	C_15_H_12_O_7_	304.0583	303.0505	303.0513	2.64	285.0406, 267.8194, 241.0521
14	Isorhamnetin	10.61	C_16_H_12_O_7_	316.0583	315.0505	315.0523	5.71	300.0289, 271.0258
15	Gentianolic acid glycoside	1.92	C_14_H_20_O_8_	316.1158	315.1080	315.1068	−3.81	153.0195, 180.0096
16	Methyl gentianolic acid glycoside	1.71	C_14_H_18_O_9_	330.0951	329.0873	329.0855	−5.47	268.9902, 191.0537, 167.0324
17	3,5-Dihydroxy-4-methyl-3-O-glucoside	2.79	C_14_H_18_O_9_	330.0951	329.0873	329.0855	−5.47	167.0350, 191.0335, 269.0456
18	Chlorogenic acid	1.33	C_16_H_18_O_9_	354.0951	353.0873	353.0843	−8.50	135.0422, 179.0329, 191.0533
19	Cryptochlorogenic acid	1.45	C_16_H_18_O_9_	354.0951	353.0873	353.0843	−8.50	173.0452, 179.0329, 191.0533
20	Methyl chlorogenate	2.79	C_17_H_20_O_9_	368.1107	367.1029	367.1031	0.54	254.0507, 178.9973, 153.0195
21	(E/Z)-Polydatin	4.50	C_20_H_22_O_8_	390.1315	389.1236	389.1235	−0.26	227.0723, 242.0100
22	Engeletin *	7.57	C_21_H_22_O_10_	434.1213	433.1135	433.1132	−0.69	287.0561, 269.0439, 259.0622, 180.0069, 151.0024
23	Isoengeletin	6.98	C_21_H_22_O_10_	434.1213	433.1135	433.1132	−0.69	287.0527, 269.0439, 259.0589, 180.0041, 151.0024
24	Quercitrin *	6.33	C_21_H_20_O_11_	448.1006	447.0927	447.0909	−4.03	301.0370, 300.0254, 271.0225, 243.0241, 151.0049
25	Cynaroside	6.76	C_21_H_20_O_11_	448.1006	447.0927	447.0909	−4.03	285.0406, 208.9391
26	Dihydrocaphen 5-O-β-D-glucoside	2.33	C_21_H_22_O_11_	450.1162	449.1084	449.1065	−4.23	287.0561, 269.0439, 153.0195
27	Dihydrocaphen 7-O-β-D-glucoside	5.05	C_21_H_22_O_11_	450.1162	449.1084	449.1065	−4.23	287.0492, 269.0439, 259.0622
28	Neoastilbin	5.64	C_21_H_22_O_11_	450.1162	449.1084	449.1065	−4.23	303.0477, 285.0406, 178.9973, 151.0376, 125.0223
29	Astilbin	6.21	C_21_H_22_O_11_	450.1162	449.1084	449.1065	−4.23	125.0223, 151.0024, 449.0978
30	Neoisoastilbin	6.67	C_21_H_22_O_11_	450.1162	449.1084	449.1065	−4.23	303.0513, 285.0406, 178.9973, 151.0376, 125.0223
31	Isoastilbin	6.83	C_21_H_22_O_11_	450.1162	449.1084	449.1065	−4.23	303.0513, 285.0406, 178.9973, 151.0376, 125.0223
32	Taxifolin 3-O-glucoside	3.81	C_21_H_22_O_12_	466.1111	465.1033	465.1027	−1.29	376.0114, 303.0121, 241.0045
33	Rutin *	4.90	C_27_H_30_O_16_	610.1534	609.1456	609.1484	4.60	300.0289, 301.0407, 229.1165, 257.3907

* Compounds identified using reference compounds.

**Table 2:** The differentially expressed metabolites.

No.	Metabolites	RT [min]	*m*/*z*	VIP	*p*-Value	Trend (DSS/Control)	Trend (JGTC-H/DSS)
1	Phosphocholine	5.58	184.0732	1.1	0.00	↓ **	
2	Glycerophosphocholine	0.57	275.1346	1.94	0.01	↓ **	↑ **
3	PE(15:0/0:0)	5.90	438.2626	1.77	0.00	↑ ***	↓ ***
4	LysoPE(15:0/0:0)	5.95	440.2765	1.89	0.00	↑ ***	↓ ***
5	LysoPC(16:1(9Z)/0:0)	6.01	494.3238	1.05	0.02	↑ *	
6	Beta-N-Acetylglucosamine	0.58	256.0593	1.29	0.00	↑ ***	
7	Xanthine	1.52	151.0254	1.15	0.00	↑ **	
8	Methionyl-Valine	2.59	247.1129	1.13	0.03	↓ *	↑ **
9	Arginylhistidine	2.29	375.1869	1.41	0.00	↓ **	↑ **
10	Threonylhistidine	2.24	221.1031	1.98	0.04	↑ *	
11	2-Oxoarginine	0.52	154.0615	1.18	0.02	↓ *	↑ ***
12	Tyrosyl-Proline	2.03	279.1336	1.59	0.00	↓ **	↑ *
13	L-phenylalanyl-L-proline	3.20	263.1387	1.70	0.00	↓ **	
14	Tyrosylhydroxyproline	3.05	293.1147	1.22	0.01	↓ **	↑ ***
15	5-(Galactosylhydroxy)-L-Lysine	1.89	347.1445	1.35	0.02	↑ *	
16	11-Ketotestosterone	4.32	366.2019	1.38	0.00	↓ ***	
17	Tetrahydrocortisol	5.43	384.2739	2.35	0.01	↑ *	↓ *
18	Betamethasone 17-benzoate	1.58	529.2594	3.09	0.00	↓ ***	↑ **
19	Cadaverine	0.46	103.1233	5.31	0.00	↑ ***	↓ ***
20	Bufexamac	2.21	206.1174	1.75	0.02	↑ *	↓ **
21	Proparacaine	2.21	277.1906	2.79	0.00	↑ **	↓ **
22	Gamma-Glutamylleucine	3.01	225.1234	4.76	0.00	↑ ***	↓ ***
23	6-Hydroxymelatonin glucuronide	4.69	457.1823	2.52	0.00	↑ ***	↓ ***
24	Tetradecanedioic acid	3.90	300.2165	3.53	0.00	↑ ***	↓ ***
25	Phenylalanylhydroxyproline	2.58	261.1232	3.07	0.00	↑ ***	↓ **
26	4-Hydroxybenzyl isothiocyanate rhamnoside	2.25	276.0686	2.90	0.00	↑ ***	↓ **
27	1′-Hydroxybufuralol	2.20	295.2013	2.05	0.01	↑ **	↓ **
28	Trans-Cinnamyl alcohol	2.01	291.1335	3.54	0.00	↑ ***	↓ ***
29	5-Aminopentanal	1.75	244.2018	3.86	0.00	↑ ***	↓ **
30	N-Cyclohexylformamide	1.03	145.1335	2.74	0.00	↑ ***	↓ ***
31	Amphetamine	2.93	153.1386	4.51	0.00	↑ ***	↓ ***
32	Sepiapterin	0.58	272.0548	3.44	0.00	↑ ***	↓ ***
33	3-Pyroglutamylthiazolidine-4-carboxylic acid	1.08	281.0015	2.88	0.00	↑ ***	↓ ***
34	Asparaginyl-Proline	1.85	274.1045	2.93	0.01	↑ ***	
35	(−)-Slaframine	2.87	243.1350	2.42	0.00	↑ ***	↓ ***
36	17-Dimethylaminogeldanamycin	4.59	597.3257	3.64	0.00	↑ ***	↓ ***
37	Buparlisib	5.13	455.1687	2.49	0.00	↑ **	↓ **
38	7alpha-Hydroxy-3-oxo-4-cholestenoate	5.91	429.2972	1.56	0.00	↑ ***	↓ **
39	N-Acetyl-4-O-acetylneuraminic acid	1.23	352.1233	3.28	0.00	↓ ***	↑ **
40	Olopatadine n-oxide	3.01	371.1959	2.98	0.04	↓ *	↑ *
41	Sakuranetin	5.17	287.0910	2.21	0.01	↓ **	↑ **
42	Asparagoside A	5.68	543.3631	2.51	0.01	↓ **	↑ *
43	Agavoside A	5.64	557.3428	2.17	0.00	↓ ***	↑ ***
44	4-Deacetylneosolaniol	2.01	341.1563	2.87	0.00	↓ ***	↑ ***
45	Linatine	0.56	242.1133	5.62	0.00	↓ ***	↑ ***
46	Dehydrovomifoliol	5.49	240.1592	2.63	0.03	↓ *	↑ ***
47	Ethyl hydrogen sulfate	0.58	170.9962	2.76	0.00	↓ **	↑ **
48	Chlorogenoquinone	1.29	333.0594	2.53	0.00	↓ **	↑ ***
49	11′-Carboxy-alpha-chromanol	5.89	439.2850	3.04	0.00	↓ ***	↑ ***
50	Dihydrogenistein	5.60	271.0614	1.86	0.01	↓ **	↑ **
51	2-Acetamido-2,6-dideoxygalactose	3.25	614.2792	3.04	0.02	↓ *	↑ ***
52	Aminodeoxykanamycin	3.07	504.2315	2.61	0.04	↓ *	↑ **
53	2,3-Dihydroxycarbamazepine	2.26	305.0339	2.60	0.00	↓ ***	↑ **
54	Niridazole	0.55	259.0130	3.01	0.00	↓ ***	↑ ***
55	Flufenamic acid	2.51	607.1285	3.76	0.00	↓ ***	
56	2-Hydroxyestrone-1-S-glutathione	5.73	633.2546	3.28	0.00	↑ **	↓ *
57	Arabinogalactose	1.80	293.0882	2.59	0.00	↓ ***	
58	Isobutyl decanoate	5.81	209.1907	2.46	0.00	↑ ***	↓ **
59	Thymidine	3.43	275.1235	2.39	0.01	↑ *	↓ *
60	Testosterone 17b-(b-D-glucuronide)	4.34	501.1882	2.14	0.00	↓ ***	↑ ***
61	4-Guanidinobutanoic Acid	0.92	146.0924	2.76	0.00	↑ ***	
62	N(omega)-Hydroxyarginine	2.94	227.0559	2.60	0.00	↑ ***	
63	Dodecanoic acid	5.65	471.3091	1.18	0.02	↑ *	↓ *
64	3-Oxododecanoic acid	4.01	259.1552	1.41	0.00	↑ **	↓ *
65	LysoPC(14:1(9Z)/0:0)	4.05	486.2571	2.39	0.00	↓ ***	
6	2-Hydroxyestrone	2.25	595.3079	3.58	0.00	↑ ***	
67	Palmitoleic acid	5.91	567.4630	2.35	0.01	↓ **	↑ **
68	Testosterone glucuronide	6.21	506.2777	2.14	0.02	↓ *	
69	7alpha,17beta-Dihydroxyandrost-4-en-3-one	5.25	349.2022	1.49	0.00	↑ ***	
70	3-Deoxyestrone	6.04	299.1386	1.57	0.01	↓ *	↑ **
71	2-Methoxyestrone 3-glucuronide	5.36	457.1846	2.18	0.00	↑ ***	↓ *
72	Choline Phosphate	5.58	184.0732	2.71	0.03	↓ *	

*↑*: increase, *↓*: reduce. * *p* < 0.05, ** *p* < 0.01, *** *p* < 0.001.

## Data Availability

The original contributions presented in this study are included in the article. Further inquiries can be directed to the corresponding author.

## Abbreviations

The following abbreviations are used in this manuscript:

UC	Ulcerative colitis
JGTC	Jingangteng capsule
SC	*Smilax china* L., Smilacaceae
DAI	Disease activity index
DSS	Dextran sulfate sodium
UPLC-QTOF-MS	Ultra performance liquid chromatography-quadrupole time-of-flight mass spectrometry
PPI	Protein–protein interaction
GO	Gene Ontology
KEGG	Kyoto Encyclopedia of Genes and Genomes
PCA	Principal component analysis
PCoA	Principal coordinate analysis
NMDS	Non-metric multidimensional scaling
LEsFe	Linear discriminant analysis effect size
PLS-DA	Partial least squares discriminant analysis
OPLS-DA	Orthogonal partial least squares discriminant analysis
AR	Androgen receptor
ChoP	Choline phosphate
L6C	*Ligilactobacillus plantarum*
CC	Cellular component
MF	Molecular function
BP	Biological process
HE	Hematoxylin and eosin
AB	Alcian blue
IL-6	Interleukin-6
IL-10	Interleukin-10
IL-1β	Interleukin-1 beta
TNF-α	Tumor necrosis factor-alpha
SOD	Superoxide dismutase
MDA	Malondialdehyde
MPO	Myeloperoxidase
D-LA	D-lactic acid
DEMs	Differentially expressed metabolites
AA	Amino acid
GPC	Glycerophospholipid
PC	Phosphatidylcholine
PA	Phosphatidic acid

## Appendix A

**Table A1:** Data from UPLC-QTOF/MS/MS on the major JGTC components in positive-ion mode.

Peak No.	Identification	RT [min]	Chemical Formula	Neutral Mass	Calculated[M-H]-(*m*/*z*)	Observed[M-H]-(*m*/*z*)	Error (ppm)	MS/MS
1	4-Hydroxybenzoic acid	2.07	C_14_H_12_O_3_	138.0317	139.0395	139.0399	2.88	98.9756, 111.0424
4	Resveratrol	7.73	C_14_H_12_O_4_	228.0786	229.0865	229.0874	3.93	103.0537, 117.0870, 135.0444
5	Oxyresveratrol	6.02	C_14_H_12_O_4_	244.0736	245.0814	245.0815	0.41	201.0499, 140.9186
6	trans-Piceatannol	5.68	C_15_H_12_O_5_	244.0736	245.0814	245.0815	0.41	140.9186, 84.9607
7	Naringenin	10.09	C_15_H_10_O_6_	272.0685	273.0763	273.0772	3.30	119.0479, 91.0567, 147.0444
8	Kaempferol	10.36	C_15_H_12_O_6_	286.0477	287.0556	287.0548	−2.79	153.0194, 137.0229, 121.0297
10	Dihydrokaempferol	6.98	C_14_H_18_O_9_	288.0634	289.0712	289.0726	4.84	140.9162, 242.9239
16	Methyl gentianolic acid glycoside	1.67	C_14_H_18_O_9_	330.0951	331.1029	331.1030	0.30	206.9977, 163.0374, 135.0444
18	Chlorogenic acid	1.30	C_16_H_18_O_9_	354.0951	355.1029	355.1034	1.41	206.9918, 188.9866, 163.0401
19	Cryptochlorogenic acid	1.45	C_16_H_18_O_9_	354.0951	355.1029	355.1034	1.41	163.0401, 191.0344
23	Isoengeletin	7.57	C_21_H_22_O_10_	434.1213	435.1291	435.1296	1.15	228.1530, 209.1672, 140.9186
22	Engeletin	7.45	C_21_H_22_O_10_	434.1213	435.1291	435.1296	1.15	327.0162, 140.9162
24	Quercitrin	6.76	C_21_H_20_O_11_	448.1006	449.1084	449.1084	0	214.9119, 140.9210, 90.9805
25	Cynaroside	6.95	C_21_H_20_O_11_	448.1006	449.1084	449.1084	0	287.0548, 214.9149, 140.9186
28	Neoastilbin	5.02	C_21_H_22_O_11_	450.1162	451.1240	451.1227	−2.88	343.0115, 209.1643, 114.0949
29	Astilbin	5.59	C_21_H_22_O_11_	450.1162	451.1240	451.1227	−2.88	339.0211, 191.0344, 139.9172
30	Neoisoastilbin	6.64	C_21_H_22_O_11_	450.1162	451.1240	451.1227	−2.88	287.0548, 201.0528
31	Isoastilbin	6.79	C_21_H_22_O_11_	450.1162	451.1240	451.1227	−2.88	327.0236, 287.0583, 201.0528

**Table A2:** Compounds used in JGTC for Network Pharmacology.

No.	Componentname	Identification
1	JGTC1	4-Hydroxybenzoic acid
2	JGTC2	Protocatechuic acid
3	JGTC3	Caffeic acid
4	JGTC4	Resveratrol
5	JGTC5	Oxyresveratrol
6	JGTC6	Trans-Piceatannol
7	JGTC7	Naringenin
8	JGTC8	Kaempferol
9	JGTC10	Eriodictyol
10	JGTC12	Quercetin
11	JGTC14	Isorhamnetin
12	JGTC18	Chlorogenic acid
13	JGTC19	Cryptochlorogenic acid
14	JGTC21	(E/Z)-Polydatin
15	JGTC22	Engeletin
16	JGTC23	Isoengeletin
17	JGTC26	Dihydrocaphen 5-O-β-D-glucoside
18	JGTC27	Dihydrocaphen 7-O-β-D-glucoside
19	JGTC28	Neoastilbin
20	JGTC29	Astilbin
21	JGTC30	Neoisoastilbin
22	JGTC31	Isoastilbin
23	JGTC32	Taxifolin 3-O-glucoside
24	JGTC33	Rutin

**Table A3:** DAI score criteria.

Score	Weight Loss	Stool Consistency	Blood in the Stool
0	None	Normal	Normal
1	1–5%	Soft but still formed	Brown
2	5–10%	Soft stool	Reddish
3	10–20%	Very soft and watery stool	Visible traces
4	>20%	Watery diarrhea	Gross rectal bleeding

**Table A4:** Colon histological score standard.

Assessment	Epithelium	Infiltration
0	Normal morphology	No infiltrate
1	Loss of goblet cells	Infiltrate around the crypt basis
2	Loss of goblet cells in large areas	Infiltrate reaching the lamina muscularis mucosae
3	Loss of crypts	Extensive infiltration reaching the lamina muscularis mucosae and thickening of the mucosa with abundant edema
4	Loss of crypts in large areas	Infiltration of the lamina submucosa
